# Recommendations of Choice of Head Coil and Prescan Normalize Filter Depend on Region of Interest and Task

**DOI:** 10.3389/fnins.2021.735290

**Published:** 2021-10-29

**Authors:** Tina Schmitt, Jochem W. Rieger

**Affiliations:** ^1^Neuroimaging Unit, School of Medicine and Health Sciences, Carl von Ossietzky Universität Oldenburg, Oldenburg, Germany; ^2^Department of Psychology, School of Medicine and Health Sciences, Carl von Ossietzky Universität Oldenburg, Oldenburg, Germany; ^3^Cluster of Excellence Hearing4all, Carl von Ossietzky Universität Oldenburg, Oldenburg, Germany

**Keywords:** 20-channel head coil, 64-channel head coil, prescan normalize, echo-planar imaging, anatomical images, MRIQC

## Abstract

The performance of MRI head coils together with the influence of the prescan normalize filter in different brain regions was evaluated. Functional and structural data were recorded from 26 participants performing motor, auditory, and visual tasks in different conditions: with the 20- and 64-channel Siemens head/neck coil and the prescan normalize filter turned ON or OFF. Data were analyzed with the MRIQC tool to evaluate data quality differences. The functional data were statistically evaluated by comparison of the β estimates and the time-course signal-to-noise ratio (tSNR) in four regions of interest, i.e., the auditory, visual, and motor cortices and the thalamus. The MRIQC tool indicated a better data quality for both functional and structural data with the prescan normalize filter, with an advantage for the 20-channel head coil in functional data and an advantage for the 64-channel head coil in structural measurements. Nevertheless, recommendations for the functional data regarding choice of head coils and prescan normalize filter depend on the brain regions of interest. Higher β estimates and tSNR values occurred in the auditory cortex and thalamus with the prescan normalize filter, whereas the contrary was true for the visual and motor cortices. Due to higher β estimates in the visual cortex in the 64-channel head coil, this head coil is recommended for studies investigating the visual cortex. For most of the research questions, the 20-channel head coil is better suited for functional experiments, with the prescan normalize filter, especially when investigating deep brain areas. For anatomical studies, the 64-channel head coil seemed to be the better choice.

## Introduction

For (functional) magnetic resonance imaging (fMRI), a variety of head coils are available with various geometries and different numbers of channels. In the current study, we investigated the differences in performance between the commercially available 20-channel and 64-channel head coils provided by Siemens for 3T MRI machines together with the influence of the prescan normalize filter in an experimental fMRI setting using three different tasks known to activate different areas in the brain with various distances from the head coil surfaces.

Coils can be characterized by different measures such as the signal-to-noise ratio (SNR), which increases with increasing number of channels but also decreases with increasing distance to the coil surface (Zwart et al., 2004; Wiggins et al., 2006, 2009; Albrecht et al., 2010; Keil et al., 2013; Reiss-Zimmermann et al., 2013). There are different ways to calculate SNR. One simple method calculates the SNR as the mean signal intensity in a region of interest (ROI) divided by the standard deviation (SD) of the noise in a background ROI (Henkelman, 1985; Kaufman et al., 1989; Constantinides et al., 1997). However, this method has the limitation that it can only be applied for non-accelerated images, because in parallel image reconstruction, noise is highly variable across the field of view (FOV) and SNR measurements with multichannel coils consequently have to consider the spatial variation in SNR. Thus, a second method estimates noise by differencing images (temporal filtering). There are two different methods described by Reeder et al. (2005): the “multiple acquisition method” and the “difference method.” In the “multiple acquisition method,” multiple images with the identical acquisition protocol are measured. The mean and the SD of each pixel are calculated over time to measure the local SNR distribution. Due to the multiple images, this method is very precise and robust, but also very time-consuming. The “difference method” (Sijbers et al., 1998) measures two identical images. The mean is obtained from a ROI from the sum of the two images, and the SD is obtained from the difference of the two images within the same ROI. As this approach is able to measure local noise, within a defined ROI, it is also very precise, even despite the small number of samples, and can be used with parallel imaging as well. Reeder et al. (2005) showed that both methods produce similar results. However, these methods do not differentiate between thermal background noise and physiological fluctuations. A third method employs high-pass filtering. According to Wilde et al. (1997), noise is estimated after spatial differentiation to eliminate the signal in homogeneous regions. This noise estimation is local and may be used in parallel imaging, but with limited accuracy. A fourth method, described by Pruessmann et al. (1999), calculates the SNR for accelerated images by using the SNR of a non-accelerated image divided by the geometry factor, which reflects a coil-dependent noise amplification across the image, and the square root of the acceleration factor. A fifth method, the pseudo multiple replica method described by Robson et al. (2008) uses a Monte Carlo simulation to produce a synthetic random noise, which is added to the acquired k-space image before the image reconstruction. This method can be used when the direct calculation of the noise is not possible and can also be applied with accelerated methods. This method allows a robust estimation of the SNR and a pixel-by-pixel image of the noise. A more general approach applicable for parallel imaging was suggested by Kellman and McVeigh (2005), who described a method for image reconstruction in SNR units on a pixel base. They estimated the noise statistics from a noise-only image (i.e., acquired without radiofrequency (RF) pulses) automatically acquired prior to signal acquisition. This method has high precision, because it acquires a large number of samples in a short time. Furthermore, the estimates of the noise covariance between channels can be used for optimizing the array combinations to improve SNR. Within the current study, we used the MRIQC toolbox to evaluate the SNR of the functional and the structural images to allow the readers to easily replicate our analyses at their own MRI machines. As implemented there, the SNR is calculated by dividing the mean intensity in the foreground ROI by the standard deviation of the same region.

In functional MRI, the time course SNR (tSNR) is also important. tSNR is calculated as the ratio of the mean signal intensity of, e.g., a voxel divided by the standard deviation of the voxel’s time series at different time points (Triantafyllou et al., 2011). It captures physiological noise, which introduces signal variations with certain temporal dynamics. Physiological noise makes a large part of the total noise, especially in 3T systems, when a medium or low spatial resolution is used (Krüger and Glover, 2001; Triantafyllou et al., 2005). Thus, tSNR is a rather accurate predictor of blood oxygenation level dependent (BOLD) detectability (Parrish et al., 2000). However, Triantafyllou et al. (2011) showed that acceleration methods can make thermal noise dominant over physiological noise.

One of the first systematic group studies investigating the advantages of multichannel coils with commonly used fMRI settings was done by Kaza et al. (2011) with the Siemens 12-channel and 32-channel head coils. Since these authors were interested in the improvements in fMRI sensitivity, they also measured the tSNR. Kaza et al. (2011) found an improvement for the 32-channel head coil compared with the 12-channel head coil. There are only two studies investigating the differences between the 20-channel and 64-channel Siemens head coils, but not with commonly used fMRI settings, as in the current study, but regarding (t)SNR in different types of acceleration methods (Seidel et al., 2020; Sica et al., 2020). According to these studies, the 64-channel head coil showed in general less tSNR decrease, as a consequence of acceleration methods, than did the 20-channel head coil.

Beside studies comparing the (t)SNR to evaluate different head coils, there are some studies comparing the BOLD signal in different tasks. Using a finger tapping paradigm, Albrecht et al. (2010) found activation in the contralateral precentral gyrus with both head coils (8-channel and 32-channel), but additional activation in other brain regions only with the 32-channel head coil. A similar finger tapping paradigm was used by Kaza et al. (2011), resulting in higher functional activation with the 32-channel head coil compared with the 12-channel head coil for cortical regions but not subcortically. Using a visual checkerboard pattern, Kahn et al. (2007) found an improvement of the BOLD signal in the 32-channel compared with a 12-channel head coil, mainly in the occipital cortex.

The number of channels within the head coil influenced not only the (t)SNR and the BOLD effects in fMRI data but also the anatomical images. Panman et al. (2019) found differences in gray and white matter volume between head coils, related to an altered tissue segmentation because of different image contrasts.

**FIGURE 1:**
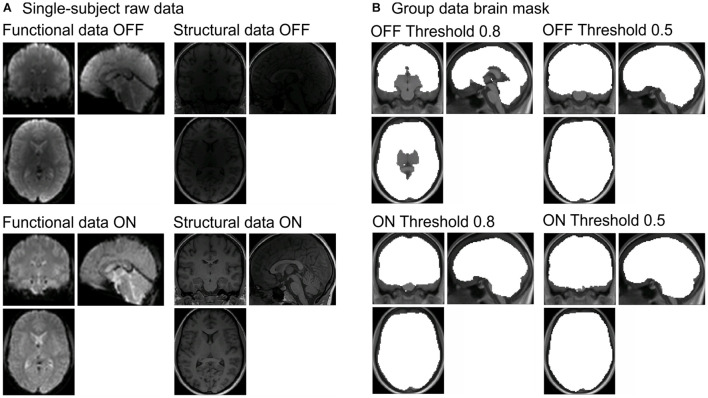
**(A)** Raw data for functional (left column) and structural data (right column) with the prescan normalize filter OFF (first row) and ON (second row) from one participant. **(B)** Differences in brain masks for the different masking thresholds (0.8 = original in left column and 0.5 = adjusted in right column) between prescan normalize OFF (first row) and ON (second row). Brain masks are created during the whole-brain analyses with SPM. SPM, Statistical Parametric Mapping.

In line with the previous paragraphs, there is an advantage of using coils with more channels utilizing smaller loops, which are closer to the participants’ head, because they result in a higher SNR. However, the disadvantage is a non-uniformity of the signal, because the depth of penetration of coils is inversely proportional to their diameters. Phased array coils have a stronger B1 sensitivity near the surface resulting in an inhomogeneous reception pattern and a sensitivity loss at larger distances from the coil surface (Roemer et al., 1990). Consequently, signals from the cortex are accentuated, while those deeper in the brain, in the subcortical regions, are attenuated (Wiggins et al., 2006) (see Figure 1A, structural and functional images labeled OFF). The resulting bright surface signal, which even counteracts the central brightening artifact often seen in 3T MRI images, is sometimes called surface coil “flare” (Roemer et al., 1990; Bernstein et al., 2006). Thus, with higher numbers of channels in receiving arrays, the intensity increase became so prominent that B1-receive-field correction became desirable. There are two ways to overcome this effect: prospective and retrospective methods (see Vovk et al., 2007, for a detailed overview of the different methods). Retrospective methods applied to the already reconstructed images can correct for low spatial frequency intensity variations, reduce noise by different filtering methods, or enhance the image contrast (Velthuizen et al., 1998; Arnold et al., 2001; Belaroussi et al., 2006). However, those are not the focus of the current work.

For multichannel parallel imaging, there is the possibility to use prospective methods for the correction of the non-uniform receiver coil profiles. Thereby, an image, used for the correction, is measured *before* the actual images are measured and a correction algorithm is applied to the actual images after their acquisition. Most manufacturers offer such a method for surface coil inhomogeneity correction, which is based on a method suggested by Brey and Narayana (1988); Narayana et al. (1988) or a variation of it. The exact implementations are proprietary and therefore not publicly available. The different vendors use different names for that procedure, i.e., prescan normalize (Siemens), Constant Level Appearance (CLEAR, Philips), or Phased array Uniformity Enhancement (PURE, GE) (Bernstein et al., 2006).

Brey and Narayana (1988) and Narayana et al. (1988) proposed a technique that combines two different approaches to compensate for the surface coil intensity loss: the *correction matrix* (Axel, 1984; McVeigh et al., 1986) and the *digital filtering* (Haselgrove and Prammer, 1986; Lufkin et al., 1986). The *correction matrix* method uses an image acquired with the surface coil, e.g., the head coil (in the following called “surface coil image”) divided by the image of a uniform phantom, i.e., a map of the surface coil intensity, to calculate the surface coil profile. The surface coil image is then divided by the calculated surface coil profile and consequently corrected for variations of the surface coil sensitivity. However, this correction matrix needs to be calculated for each specific position and surface coil. Instead of measuring a surface coil image and dividing that by the image of a phantom, with the *digital filtering* method, the surface coil profile is obtained by smoothing the image with appropriate digital filter. This method can also be used when the surface coil profile is unknown. However, the disadvantage here is that the filtering assumes that the surface coil intensity varies more slowly than any feature of interest. Especially when using a small FOV, this method cannot provide a true correction or surface coil sensitivity since large areas of the images often vary in intensity. Thus, Brey and Narayana proposed a procedure that combines both methods to eliminate the limitations of determining the position of the surface coil on the one hand and make assumptions about the information of the image on the other hand.

Brey and Narayana (1988) developed a correction method in which for each image, prior to the actual measurement (“prescan”), a surface coil profile is determined using a surface coil image and a body coil image over the maximum FOV. Surface coils usually have a high SNR but severe intensity inhomogeneities, whereas body coil images are assumed to be more homogeneous but with poorer SNR. The method described by Brey and Narayana (1988) and Narayana et al. (1988) allows to obtain an intensity inhomogeneity corrected image of the surface coil with a high SNR. A first step is the construction of the complex phase images from the surface and the body coil raw data. As both are noisy, the next step includes the spatial smoothing of both. The reciprocal of the surface coil profile, calculated by dividing the smoothed (low-pass filtered) body coil image by the smoothed surface coil image, can then be used as a correction matrix. By multiplying the uncorrected surface coil image with the magnitude of the smoothed reciprocal profile, the corrected surface coil image can be calculated, which will preserve the SNR of the surface coil but with its inhomogeneity removed (Narayana et al., 1988) (see Figure 1A, functional and structural images labeled ON). This method provides a correction of the sensitivity profile of surface coils but does not specify how to combine images of different coil arrays.

With multichannel arrays, there are multiple ways to combine the signals from the different coils. One possibility is the sum-of-square method to combine images from different arrays. It assumes that the signal variation over voxels represents sensitivity and uses the square root of the sum of squares of the voxel values from the different coils to combine them. Although this method is quite simple, fast, and stable, it is sub-optimal in regions with low SNR and, more importantly, during the reconstruction, the phase information will be lost. A better approach to combine the signals of different arrays, using a complex reconstruction, is described by Roemer et al. (1990) where they combined the signal as a pixel-by-pixel sum of the coil signals, weighted by the individual coil sensitivity at the location of that pixel. However, for that approach, the coil sensitivity for the individual coils needs to be known. This is theoretically the most accurate method but may lead to a mis-registration between prescan and the actual imaging. The high resolution of the prescan might lead to lower SNR, which makes a post-processing necessary (Pruessmann et al., 1999). With an adaptive coil combination as proposed by Walsh et al. (2000), coil sensitivities can be calculated directly from the complex images by using an eigenvector analysis of the local data covariance matrix, which preserves the phase information and a good SNR, as the information is directly estimated from the imaging data. However, with that approach, an absolute reference for the phase is missing, leading to phase singularities. Thus, starting from Siemens software version VE11, their prescan normalize filter uses a calibration scan with the body coil to obtain a phase reference image and to calculate the low-resolution estimates of the surface coil sensitivities relative to the body coil. This phase reference image is used to correct the surface coil image. Afterward, the adaptive coil combination is done with the surface coil sensitivity estimation directly from the phase-corrected complex data (Jellus and Kannengiesser, 2014). One of the advantages of that approach is that it avoids phase artifacts, as the body coil phase is the best available phase reference. It also allows to find the best coil combination coefficients, and it might be less sensitive for spatial mis-registration. In contrast to the sum-of-square method, the individual sensitivity at each pixel is considered, and the qualitatively better phase images are used as a basis for the calculation of the correction factor. Note that the adaptive coil combination is used during the calculation of prescan normalize filter, while this filter is then applied to the functional measurements, and the latter are reconstructed by using GRAPPA. Thus, the prescan normalize filter might affect not only the signal intensity but also the tSNR. This has already been shown by Kaza et al. (2011), where they found an increase in tSNR with both coils when using the prescan normalize filter.

One of the major limitations of this method is the requirement of the “prescan” prior to the imaging. Especially in functional MRI, where long series of images are acquired, motion is a major problem. If there is any motion of the participant, either between the prescan and the actual image acquisition, or severe motion during a longer image acquisition, the correction fails or generates artifacts. Furthermore, an reviewer provided the hint that prescan normalize will not be compatible with the option “Matrix optimization: Performance,” which is mandatory for very long experiments, since otherwise the reconstruction may abort.

So far, we are not aware of literature providing clear recommendation when to use this prescan normalize filter. Within the original echo-planar imaging (EPI) sequence provided by Siemens in our 3T Prisma (VE11C), the prescan normalize filter is turned OFF. However, in previous studies, we found signal drops especially in the subcortical regions (Figure 1B, labeled OFF Threshold 0.8) when analyzing the data using the standard Statistical Parametric Mapping (SPM) algorithms. Especially in studies focusing on subcortical brain structures, the default detection threshold for the brain mask was too conservative. When changing the masking threshold in SPM to 0.5 instead of 0.8, it was possible to get those subcortical brain structures into the brain mask (Figure 1B, labeled OFF Threshold 0.5). Instead of changing the masking threshold, the usage of the prescan normalize filter also allows the brain mask to include the subcortical brain structures. Besides the study by Kaza et al. (2011), we could not find any literature reporting results about the consequences of using the prescan normalize filter. They found higher tSNR with the prescan normalize filter turned ON in the 32-channel head coil. According to their results, filtering did not affect the overall magnitude of the activation in the 12-channel head coil; however, there was an improvement in tSNR in the 32-channel head coil for the subcortical and cerebellar areas.

In the systematic fMRI group-level study presented here, we investigated the effects of the different head coils together with the prescan normalize filter in commonly used fMRI settings. In contrast to the study by Kaza et al. (2011), we used three different tasks, resulting in robust activations of different parts of the brain, i.e., the auditory, motor, and visual cortices and deep brain areas such as the thalamus. In addition to the standard analyses of fMRI data, such as first- and second-level analyses, and calculation of tSNR, we systematically assessed the effect of the different head coils and the prescan normalize filter with the MRIQC tool provided by Esteban et al. (2017). This toolbox allows the analyses of different data quality measures calculated from the functional and structural data. With these types of analyses, we seek to find outstanding differences between head coils with and without the prescan normalize filter.

## Materials and Methods

### Participants

We investigated 26 right-handed healthy volunteers (12 males and 14 females; age range 19–31 years; average age (mean ± SD): 24.6 ± 2.8 years). The study was approved by Ethics Committee of the University of Oldenburg (Kommission für Forschungsfolgenabschätzung und Ethik) and was carried out in accordance with the Declaration of Helsinki and with the EU general data protection regulation. Written informed consent was obtained from all participants. Analyses with the MRIQC tool included the data of all participants. Two participants were removed from all further analyses due to significant head movements during at least one of the sessions. One additional participant was removed due to non-compliance with the task instruction. Thus, 23 participants remained for further statistical analyses of functional data.

### Head Coils

Two commercially available MRI head coils were compared. The 20-channel head/neck coil consists of 20 loops arranged on two rings with eight elements each and one ring with four elements. The head coil had an inner vertical diameter of 26.5 cm and an inner horizontal diameter of 23 cm. The 64-channel head/neck coil consists of an anthropomorphic geometry with 64 loops, whereas the upper coil part consists of 24 elements and the lower coil part of 40 elements. The head coil had an inner vertical diameter of 22 cm and an inner horizontal diameter of 19.5 cm. Although both coils are head/neck coils, throughout the manuscript, we will refer to them as “head coils.”

### Experimental Procedure

Three different tasks were used as experimental conditions, i.e., motor, auditory, and visual tasks. Participants performed all tasks while lying in supine position in the scanner with their head comfortably fixed with cushions in order to avoid involuntary movement. All visual stimuli were presented in white color on a black background screen (PROPixx, VPixx Technologies Inc., Saint-Bruno, QC, Canada). In each task, a 20 s stimulus block was followed by a 20 s rest block in which only a fixation cross was presented and the participants only had to fixate their gaze on the cross. These two blocks were repeated eight times, resulting in a total duration of 6 min. Each task was repeated twice, with the prescan normalize filter turned ON or OFF.

In the motor task, the stimulus block consisted of a sequence of 20 visual stimuli with number 2, 3, or 4; and participants had to press with their index, middle, or ring finger, respectively (response pads: Current Designs Inc., Philadelphia, PA, United States), as fast and accurately as possible. Each of the numbers was presented for 500 ms followed by 500 ms fixation cross. At the beginning of each block, there was a written instruction on which hand to use in the upcoming block. The left and right hands were alternatively used over blocks. At the end of each block, a feedback was presented including the percent correct responses as well as the mean reaction time. Each information screen lasted for 1 s followed by 1 s with the fixation cross. Before the actual motor task started, participants had the possibility to practice in a short sequence once for the left and once for the right hand without running the MRI. In the auditory task, the stimulus block consisted of five sentences, each lasting for about 4 s (presented via OptoActive head phones, OptoAcoustics Ltd., Mazor, Israel) randomly selected out of the Oldenburg corpus of Linguistically and Audiologically Controlled Sentences (OLACS; Uslar et al., 2013). Participants only had the passive task to listen to the stimuli and to fixate the cross presented on the screen throughout the whole task. In the visual task, the stimulus block consisted of a disk-shaped flickering visual checkerboard (1-Hz flicker) with a visual angle of 5°. Participants only had the passive task to view the visual checkerboard or the fixation cross presented during the rest block.

Thereafter, a structural image of the brain was acquired. This first part of the experiment lasted for about 45 min. Afterward, the head coil was changed, and the second part of the experiment started, which included the same tasks and as well as the structural image. The second structural image was necessary, because the differences in structural images between the different head coils were also compared. The whole experiment lasted for about 2 h. The order of the head coils was counterbalanced across participants.

### Data Acquisition

Magnetic resonance imaging data were acquired by a 3T Siemens Magnetom native Prisma (VE11C) whole-body MRI with the 20-channel and 64-channel Siemens head coils. Functional images were acquired using an EPI sequence with BOLD contrast (repetition time (TR) = 2,000 ms, echo time (TE) = 30 ms, flip angle = 75°, distance factor = 20%, slice thickness = 3 mm, FOV = 192 × 192 mm, matrix = 64 × 64, voxel size = 3 × 3 × 3 mm^3^, 36 slices, GRAPPA = 2, prescan normalize = ON/OFF, depending on the run). Each of the auditory and visual tasks consists of 170 volumes; the motor task consists of 185 volumes, due to the instruction and feedback at the beginning and end of each block. Structural images were acquired with a 3D T1-weighted sequence (MPRAGE, TR = 2,000 ms, TE = 2.07 ms, flip angle = 9°, voxel size = 0.75 × 0.75 × 0.75 mm^3^, GRAPPA = 2, FOV = 240 × 240, 224 sagittal slices, TA = 6:16 min). For illustration purposes, examples of the raw data for functional and structural images with prescan normalize filter turned ON and OFF are shown in Figure 1A for one participant.

### Data Analyses

#### Functional Data

##### MRIQC for functional data

In a first step, the MRIQC software (Esteban et al., 2017) was used to calculate image quality metrics (IQMs). We decided for that standard tool (without any modifications) to make the study more comparable with the current literature, although some of the IQMs are rather global and other metrics might be more sensitive to local aspects. However, that would be out of the scope of the current work and, furthermore, make the current study less comparable with the recent literature and millions of already computed IQMs^1^. The IQMs for the functional raw data are separated into three categories: (a) spatial information, (b) temporal information, and (c) artifacts and others. For each of the measures, a three-factor repeated-measures ANOVA was calculated with the factors task (motor, auditory, vs. visual), head coil (20-channel vs. 64-channel), and prescan normalize (ON vs. OFF). As a measure of spatial information, the SNR is reported, whereby higher values are better. The MRIQC tool calculates the SNR either separately for each tissue or as a total, by dividing the mean intensity in the foreground ROI, (white matter, gray matter, or cerebrospinal fluid) by the SD of the same region, although this procedure might be suboptimal regarding any spatial inhomogeneities. As a measure of temporal information, the global correlation (GCOR) is reported. For GCOR, a correlation coefficient was calculated over all possible combinations of voxels and averaged over the whole brain, i.e., the sum of pairwise voxel correlations of all voxels in the brain (Saad et al., 2013). GCOR in MRIQC is calculated by using an algorithm implemented in AFNI (Cox, 1996), which consists of three steps. According to Saad et al. (2013), there is a simple way to calculate that: each voxel’s time series is de-meaned and scaled by its Euclidean norm. Those scaled time series were averaged over the whole-brain mask. Finally, GCOR is calculated as the length (L^2^ norm) of this averaged series (Saad et al., 2013). For GCOR, lower values are better, as spatially uncorrelated noise should average out in this procedure. Although motion and physiological noise should not differentiate between head coils and prescan normalize filter state *per se*, the different tasks used in this study might also influence the GCOR and might lead to differences in the values. As a measure of imaging artifacts, the ghost-to-signal ratio (GSR) is reported, as it calculates the mean signal in the areas of the image that are prone to ghosting based on the phase encoding direction, i.e., lower values are better. The ANOVA results for all further MRIQC measures can be found in the Supplementary Material A.

##### Region of interest-based and whole brain analyses

The functional data were analyzed using the Statistical Parametric Mapping software package (SPM12, Wellcome Department of Imaging Neuroscience, London, United Kingdom) running under MATLAB 2019b (The MathWorks, Inc.). The preprocessing steps of each of 12 datasets (two head coils × two prescan normalize × three tasks) per participant consisted of a slice time correction, realignment, coregistration, and normalization to the Montreal Neurological Institute (MNI) stereotactic space by using parameters obtained from a segmentation of the corresponding structural image. All peak coordinates of the statistical analyses are reported in MNI coordinates.

In the first-level analysis, a general linear model described the different stimulus blocks (motor response, auditory stimulation, or visual stimulation) as boxcar functions within one regressor, convolved with the canonical hemodynamic response function. Head movement parameters were entered as additional regressors. A temporal high-pass filter (128 s) and an AR(1) model were applied to remove temporal autocorrelations. The masking threshold was selected such that deep brain areas were included in the masks independent of whether prescan normalize was turned ON or OFF (for differences between ON and OFF without this adjustment see Figure 1B). ROIs were defined for the motor cortex (bilateral area 4a and 4p), the auditory cortex (bilateral areas TE 1.0, TE 1.1, and TE 1.2), and the visual cortex (bilateral hOC1 and hOC2), as well as the thalamus, as described in the cytoarchitectural maps from the Anatomy toolbox (Eickhoff et al., 2005, 2006, 2007).

A ROI-based analysis was carried out on non-smoothed first-level results for each individual participant. For the second-level analysis, we extracted the contrast β estimates for the highest activated voxel in each ROI. β estimates for each participant in each condition served as input to an ANOVA with the following factors: ROI (motor, auditory, visual, vs. thalamus), head coil (20-channel vs. 64-channel), task (motor, auditory, vs. visual), and prescan normalize (ON vs. OFF) with Greenhouse–Geisser correction. Statistical data analyses were performed in SPSS (IBM SPSS Statistics 26). This analysis, which uses a spatial prior (the pre-defined ROI), has several advantages. It only relies on the highest activated voxel in each ROI, which does not need to be exactly at the same position in each participant. Thus, this approach relaxes the requirement of an accurate functional anatomical match between individual brains and the requirement for spatial smoothing to improve the spatial matching. Moreover, the statistical analysis is based on single voxels and does not require any cluster level statistical corrections, as is the case in a whole-brain analysis, where effects in many voxels are tested simultaneously. Thus, it allows to find weaker activations and does not require smoothing to control for the number of independent tests (Worsley et al., 1996). However, as the name states, the ROI analysis is restricted to the pre-defined regions and therefore misses potential effects in other brain regions.

For an overview of the activation effects, we performed a whole-brain full factorial ANOVA to reveal main effects of head coil (20-channel vs. 64-channel), task (motor, auditory, vs. visual), and prescan normalize (ON vs. OFF) as well as their interactions. In this analysis, we smoothed the normalized data with a Gaussian kernel with 8-mm full-width-half maximum to improve spatial overlap between individual activations and to control for the number of statistical comparisons (Worsley et al., 1996). Smoothing was done in the first-level analysis prior to the estimation of the βs. The individual βs of the first level analysis were then used for the full factorial ANOVA in the second-level whole-brain analysis. For the whole-brain analyses, BOLD activation effects were considered significant when they passed a corrected significance threshold of *p* < 0.05 (family wise error (FWE) corrected and a minimum cluster size of 20 voxel). The FWE correction in SPM is based on random field theory (RFT), as Bonferroni correction for the number of voxels is considered too conservative (Worsley et al., 1996). RFT accounts for the fact that spatial smoothing reduces the number of independent observations. It allows to calculate the expected Euler characteristic of a random field of *z, t*, or *F*-values with known smoothness (meaning a known number of resolution elements) and shape. The expected Euler characteristics are the expected number of voxel clusters in a random field that cross a certain threshold. For FWE correction, SPM would select a threshold *z, t*, or *F*-value such that we can expect with 5% probability that least one or more blobs in the map cross the threshold by chance. In other words, any of the blobs in the resulting thresholded map have a probability ≤ 0.05 that occurred by chance. In an additional exploratory analysis, to increase statistical sensitivity, we performed ROI analyses on the smoothed data and show the conjunction of the cytoarchitectural defined ROI and a liberal threshold of *p* < 0.001 and a minimum cluster size > 20 voxel. To further investigate the nature of the interactions revealed by the exploratory analysis, we extracted the β estimates in a sphere with 10-mm radius around the local maximum peak coordinates for each condition and each participant.

#### Time-Course Signal-to-Noise Ratio

Individual tSNR maps were calculated using an additional SPM script provided by Cyrill Pernet^2^ written on the basis of the work by Liu (2016).

To evaluate the tSNR, a ROI analysis was conducted for the same brain areas as described above, by extracting the mean tSNR in each voxel in the given ROI for each condition. The resulting data were analyzed in an ANOVA with the following factors: ROI (motor, auditory, visual, vs. thalamus), head coil (20-channel vs. 64-channel), task (motor, auditory, vs. visual), and prescan normalize (ON vs. OFF) over all participants with Greenhouse–Geisser correction. In addition, averaged tSNR maps for each condition were calculated for better visualization.

#### MRIQC for Structural Data

Comparable with the analyses of the functional data, the MRIQC software was used to calculate the IQMs for the structural raw data. The IQMs fall into four categories: (a) noise measurements, (b) information theory, (c) specific artifacts, and (d) other measures. For each of the individual measures, an ANOVA was calculated for the factors head coil (20-channel vs. 64-channel) and prescan normalize (ON vs. OFF). The SNR as a measure of noise is calculated within the respective tissue mask. The contrast-to-noise ratio (CNR) (Magnotta and Friedman, 2006), as an extension of the SNR calculation to evaluate how separated the tissue distribution of gray and white matter are, is calculated as the mean of the gray matter intensity values minus the mean of the white matter intensity values divided by the standard deviation of the values outside the brain. Therefore, higher values indicate better quality. Note that without the prescan normalize filter, there is a correlated low-frequency spatial modulation of the intensity of gray and white matter. In case gray and white matter are brighter at the cortex than in the central brain areas, by the same factor, the CNR is still constant, because the same value (SD of values outside the brain) is used for division. Thus, the CNR measure might indicate an advantage for the prescan normalize filter, even when the contrast does not really change. Nevertheless, this effect might not change potential performance differences between head coils. Results of all other measures in the MRIQC tool regarding the structural data can be found in the Supplementary Materials B.

## Results

### Functional Data

#### MRIQC for Functional Data

**FIGURE 2:**
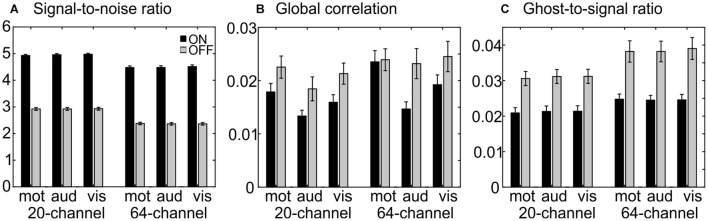
MRIQC results for the functional data: **(A)** results for the signal-to-noise ratio; higher values are better. **(B)** Results for global correlation; smaller values are better. **(C)** Results for the ghost-to-signal ratio; lower values are better. Each figure represents the different tasks (motor, auditory, and visual) separated for each head coil (blocks on the left side for the 20-channel head coil and on the right side for the 64-channel head coil) as well as the prescan normalize ON (black) and OFF (gray) condition.

The ANOVA for SNR showed a main effect of head coil, F(1,25) = 164, *p* = 1.73E–12, η^2^ = 0.868, with larger SNR for the 20-channel head coil and a main effect of prescan normalize, F(1,25) = 2616, *p* = 7.97E–27, η^2^ = 0.990, with larger SNR for prescan normalize ON and no interaction (Figure 2A).

The ANOVA for the GCOR revealed a main effect of head coil, F(1,25) = 6.51, *p* = 0.017, η^2^ = 0.206, with lower values for the 20-channel head coil, a main effect of prescan normalize, F(1,25) = 26.1, *p* = 2.76E–5, η^2^ = 0.511, with lower values for prescan normalize ON, and a main effect of task, F(2,50) = 5.98, *p* = 0.004, η^2^ = 0.193, with lower values in the auditory task (Figure 2B).

The ANOVA for the GSR in the y-direction resulted in a main effect of head coil, F(1,25) = 27.9, *p* = 1.77E–5, η^2^ = 0.527, with less ghosting artifacts in the 20-channel head coil and a main effect of prescan normalize, F(1,25) = 87.2, *p* = 1.24E–9, η^2^ = 0.777, with less ghosting artifacts in prescan normalize ON as well as an interaction between head coil and prescan normalize, F(1,25) = 8.8, *p* = 0.006, η^2^ = 0.261, with less differences between prescan normalize ON and OFF in the 20-channel head coil and larger differences in the 64-channel head coil (Figure 2C).

Regarding the functional data, all measures point to better results for the 20-channel head coil with the prescan normalize filter ON.

#### Region of Interest-Based and Whole-Brain Analyses

##### Region of interest-based β estimates

The ANOVA for β estimates revealed a main effect of ROI, F(3,66) = 164, *p* = 2.70E–20, η^2^ = 0.882, with largest β estimates for the visual (12.32), followed by the auditory (6.07) and motor cortices (5.30) and finally the thalamus (2.85); a main effect of task, F(2,44) = 26.0, *p* = 2.86E–7, η^2^ = 0.542, with larger β estimates for the visual (7.83) and motor (6.90), followed by the auditory task (5.18) and a main effect of prescan normalize, F(1,22) = 78.6, *p* = 1.02E–8, η^2^ = 0.782, with larger β estimates for prescan normalize OFF (7.33 vs. 5.95). There was no significant difference between head coils. An overview over all main effects and interactions is shown in more detail in Table 1A.

**TABLE 1:** Results for extracting β estimates in highest activated voxels.

		df	F	p	η^2^	Description
**(A) ANOVA: ROI × coil × task × prescan**						
	ROI	3,66	163	2.70E–20	0.882	
	Coil	1,22	2.84	0.106	0.115	
	Task	2,44	26.0	2.86E–7	0.542	
	Prescan	1,22	78.6	1.02E–8	0.781	
	ROI × coil	3,66	11.6	1.35E–5	0.346	Increase in vis ROI from 20- to 64-channel; other ROIs either stay the same or decrease.
	ROI × task	6,132	141	1.59E–19	0.866	Larger values in ROIs involved in task, e.g., aud task activates aud ROI.
	Coil × task	2,44	1.98	0.151	0.083	
	ROI × coil × task	6,132	0.85	0.492	0.037	
	ROI × prescan	3,66	87.5	5.30E–16	0.799	Larger values with prescan OFF in vis and mot ROI.
	Coil × prescan	1,22	1.10	0.304	0.048	
	ROI × coil × prescan	3,66	4.92	0.020	0.183	See Table 1B for interaction.
	Task × prescan	2,44	34.9	1.03E–9	0.613	Larger values with prescan OFF in vis and mot task.
	ROI × task × prescan	6,132	22.8	1.71E–10	0.509	See Table 1C for interaction.
	Coil × task × prescan	2,44	1.25	0.291	0.054	
	ROI × coil × task × prescan	6,132	2.52	0.060	0.103	
**(B) ANOVA: ROI × coil × prescan**						
Visual	Coil	1,22	17.7	4.00E–4	0.446	64-channel (13.23) > 20-channel (11.41)
	Prescan	1,22	128	1.20E–10	0.854	OFF (14.77) > ON (9.87)
	Coil × prescan	1,22	4.71	0.041	0.176	Figure 3B (visual)
Motor	Coil	1,22	0.01	0.933	0.0003	
	Prescan	1,22	46.3	7.75E–7	0.678	ON (6.61) > OFF (4.00)
	Coil × prescan	1,22	0.29	0.593	0.013	Figure 3B (motor)
Auditory	Coil	1,22	2.63	0.119	0.107	
	Prescan	1,22	5.17	0.033	0.190	ON (6.27) > OFF (5.88)
	Coil × prescan	1,22	4.36	0.048	0.166	Figure 3B (auditory)
Thalamus	Coil	1,22	0.03	0.863	0.001	
	Prescan	1,22	90.6	2.92E–9	0.805	ON (3.65) > OFF (2.05)
	Coil × prescan	1,22	6.56	0.018	0.230	Figure 3B (thalamus)
**(C) ANOVA: ROI × task × prescan**						
Visual	Task	2,44	99.0	1.32E–11	0.818	vis (21.46) > mot (9.56) > aud (3.21)
	Prescan	1,22	60.3	9.57E–8	0.733	OFF (13.42) > ON (9.40)
	Task × prescan	2,44	21.0	1.87E–5	0.489	Figure 3A (visual)
Motor	Task	2,44	48.9	5.09E–9	0.690	mot (8.77) > vis (3.84) > aud (3.35)
	Prescan	1,22	34.0	7.22E–6	0.607	OFF (6.71) > ON (3.93)
	Task × prescan	2,44	3.81	0.037	0.148	Figure 3A (motor)
Auditory	Task	2,44	127	2.18E–19	0.854	aud (12.21) > mot (4.06) > vis (2.48)
	Prescan	1,22	17.7	3.00E–4	0.447	ON (6.61) > OFF (5.89)
	Task × prescan	2,44	7.39	0.002	0.252	Figure 3A (auditory)
Thalamus	Task	2,44	11.5	3.00E–4	0.344	mot (3.73) > vis (2.89) > aud (1.98)
	Prescan	1,22	41.5	1.73E–6	0.654	ON (3.49) vs. OFF (2.24)
	Task × prescan	2,44	0.73	0.431	0.032	Figure 3A (thalamus)

***(A)** Results of the four-factorial ANOVA, **(B)** separate analyses for each ROI for the ROI × head coil × prescan normalize interaction, and **(C)** separate analyses for each ROI for the ROI × task × prescan normalize interaction. ROI, region of interest. vis = visual task/ROI, aud = auditory task/ROI, mot = motor task/ROI.*

**FIGURE 3:**
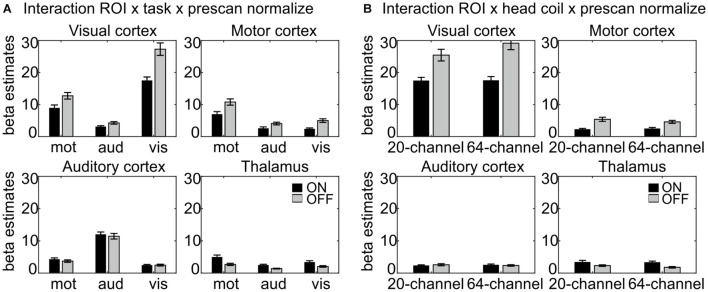
Results of the interactions **(A)** ROI × task × prescan normalize and **(B)** ROI × head coil × prescan normalize, separated for each ROI. **(A)** Largest β estimates in each ROI correspond to the respective task. Note that participants saw numbers on the screen in the motor task, thus resulting in enhanced β estimates in the visual cortex also for the motor task. There were differences regarding the prescan normalize filter with larger β estimates for prescan normalize OFF (gray) in the visual and motor cortices, whereas there was no difference between prescan normalize ON (black) and OFF in the auditory cortex and even the contrary pattern with larger values for prescan normalize ON in the thalamus. **(B)** Regarding the differences in the head coils, there were larger β estimates for the prescan normalize OFF in the visual and motor cortices, whereas again, there was no difference in the auditory cortex, but larger values for prescan normalize ON in the thalamus. ROI, region of interest.

The interaction ROI × task × prescan normalize, F(6,132) = 22.8, *p* = 1.71E–10, η^2^ = 0.509 (Figure 3A) indicated, that, as expected, the highest β estimates occurred in the ROI corresponding to the task; e.g., in the auditory cortex, the β estimates for the auditory task were largest. Separate analyses for each ROI were calculated and reported in detail in Table 1B and Figure 3A.

The interaction ROI × head coil × prescan normalize, F(3,66) = 4.93, *p* = 0.02, η^2^ = 0.183 (Figure 3B), indicated differences between head coils mainly in the visual cortex. Separate analyses for each ROI were calculated and reported in detail in Table 1C and Figure 3B.

##### Whole-brain analysis

**FIGURE 4:**
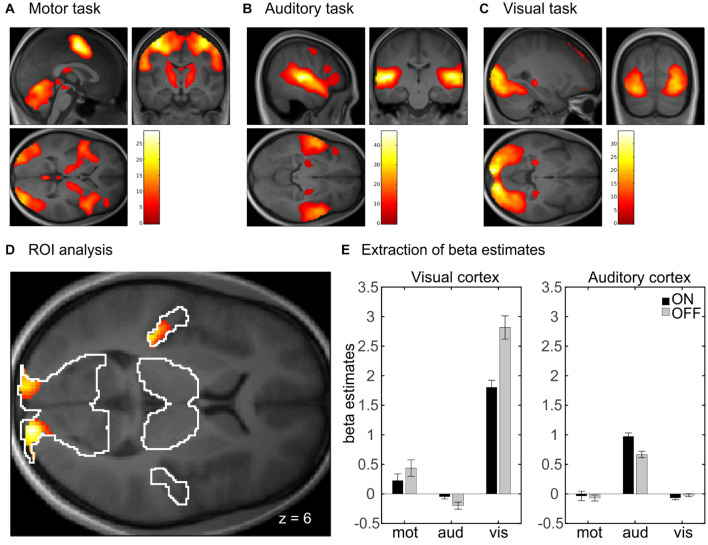
Whole-brain analyses with main effect of task separated for **(A)** motor, **(B)** auditory, and **(C)** visual tasks. Results are whole-brain FWE corrected (*p* < 0.05) with cluster level > 100. **(D)** Interaction between task and prescan normalize in whole-brain fMRI analysis. ROI analyses with activation in bilateral visual cortex and left auditory cortex, FWE corrected (*p* < 0.05). **(E)** Extraction of β estimates in peak maximum of the visual and auditory cortex. β estimates for prescan normalize OFF (gray) are larger in the visual cortex for both tasks involving visual cortex activity, i.e., the visual and the motor task. β estimates in the auditory cortex are larger for prescan normalize ON (black). FWE, family wise error; ROI, region of interest.

In the whole-brain analysis, there was no significant main effect of head coil, indicating no difference between the 20-channel and 64-channel head coils, but a main effect of prescan normalize in the bilateral visual cortex, using ROI analyses, with higher activation for prescan normalize OFF compared with ON (see Table 2A). There was a main effect of task with activations of the whole brain. This results from the fact that three different tasks were included: motor, auditory, and visual tasks. In Figures 4A–C, the main effect of task was shown separately for the three tasks to indicate that the respective brain areas are involved in each of the tasks. Results are shown with whole-brain FWE correction.

**TABLE 2:** Results of the whole-brain ANOVA for **(A)** the main effect of prescan normalize and **(B)** the interaction between task and prescan normalize.

Contrast	Brain region	[x y z] in MNI	Cluster size	*z*-value
**(A)**				
Prescan	R visual cortex	18 –100 6	242	5.17
	L visual cortex	–16 –102 12	85	4.14
**(B)**				
Prescan × Task	R visual cortex	16 –100 6	522	6.91
	L visual cortex	–10 –98 0	375	6.11
	L auditory cortex	–40 –28 6	131	5.47

*Coordinates are reported as normalized to the MNI space. MNI, Montreal Neurological Institute.*

There was also an interaction between prescan normalize and task with activations in the bilateral visual cortex and the left auditory cortex (Figure 4D and Table 2B). Extraction of the mean β estimates within a sphere of 10-mm radius around the peak maximum indicated that in the visual cortex, β estimates are larger in the visual task but also present in the motor task (because participants saw the numbers on the screen), with larger values for prescan normalize OFF (Figure 4E, visual cortex). In contrast, the peak maximum in the auditory cortex showed the largest β estimates, as expected, in the auditory task, but here, the β estimates for prescan normalize ON were larger (Figure 4E, auditory cortex).

#### Time-Course Signal-to-Noise Ratio

**FIGURE 5:**
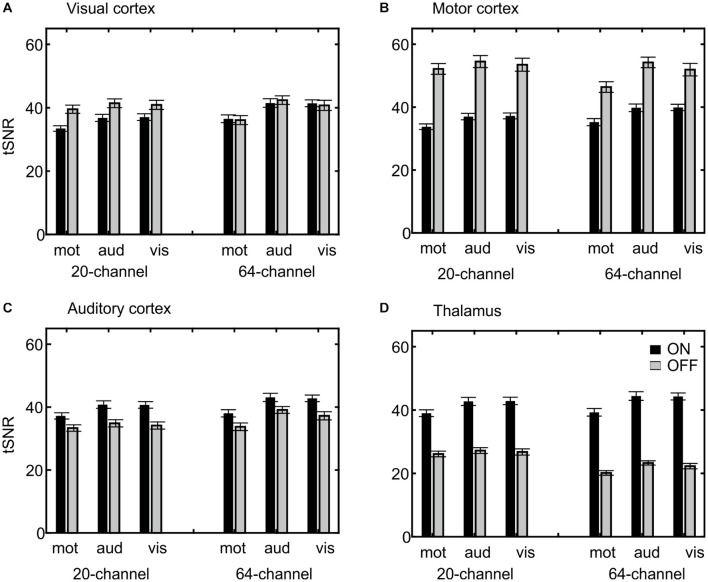
Visualization of the ROI × head coil × task × prescan normalize interaction of the tSNR ROI analyses. Individual subfigures show the results for the different ROIs separately for the different head coils (left 20-channel, right 64-channel), tasks (motor, auditory, and visual) and the prescan normalize filter ON (black) and OFF (gray). Results for the visual **(A)** and motor cortex **(B)** are comparable and in contrast to the auditory cortex **(C)** and thalamus **(D)**, with a larger tSNR for prescan normalize OFF and with stronger differences between ON and OFF in the 20-channel head coil compared with the 64-channel head coil. In the **(C)** auditory cortex, there was a higher tSNR for the 64-channel head coil and the prescan normalize ON. In contrast, the **(D)** thalamus showed the similar pattern for the prescan normalize ON but higher tSNR in the 20-channel head coil. ROI, region of interest; tSNR, time-course signal-to-noise ratio.

Extraction of the mean tSNR in each ROI revealed a main effect of ROI, F(3,66) = 237, *p* = 6.22E–25, η^2^ = 0.915, with the highest tSNR in the motor (44.64), followed by the visual (38.99) and auditory cortices (37.92) and finally the thalamus (33.20), a main effect of task, F(2,44) = 25.8, *p* = 2.34E–6, η^2^ = 0.540, with the highest tSNR in the auditory (40.19) followed by the visual (39.61) and motor task (36.25) and a main effect of prescan normalize, F(1,22) = 5.78, *p* = 0.025, η^2^ = 0.208, with higher tSNR with prescan normalize ON (39.36 vs. 38.00). An overview over all main effects and interactions is shown in more detail in Table 3A. Because the overall interaction was significant as well, follow-up ANOVAs were calculated for each ROI separately and reported in detail in Table 3B and Figure 5.

**TABLE 3:** Results for tSNR analyses.

		df	F	p	η^2^	Description
**(A) ANOVA: ROI × coil × task × prescan**						
	ROI	3,66	237	6.22E–25	0.915	
	Coil	1,22	0.46	0.502	0.021	
	Task	2,44	25.8	2.34E–6	0.540	
	Prescan	1,22	5.78	0.025	0.208	
	ROI × coil	3,66	56.8	1.13E–12	0.721	Decrease from 20-channel to 64-channel in thalamus; no difference in mot cortex but increase from 20-channel to 64-channel in aud and vis cortex.
	ROI × task	6,132	8.98	1.00E–4	0.290	Strong increase from mot to aud and vis task in all ROIs with largest values in mot cortex.
	Coil × task	2,44	3.31	0.053	0.131	
	ROI × coil × task	6,132	7.41	0.001	0.252	
	ROI × prescan	3,66	511	1.07E–22	0.959	Larger values in mot cortex with prescan OFF, but larger values in thalamus with prescan ON.
	Coil × prescan	1,22	40.8	1.95E–6	0.650	Increase in tSNR from 20-channel to 64-channel with prescan ON, but decrease from 20-channel to 64-channel with prescan OFF.
	ROI × coil × prescan	3,66	29.8	4.07E–7	0.576	
	Task × prescan	2,44	1.90	0.164	0.079	
	ROI × task × prescan	6,132	14.3	9.57E–6	0.394	
	Coil × task × prescan	2,44	1.12	0.332	0.049	
	ROI × coil × task × prescan	6,132	5.64	0.004	0.204	Figure 4, see Table 3B for interaction
**(B) ANOVA: ROI × task × prescan**						
Visual	Coil	1,22	4.94	0.037	0.184	64 (39.77) > 20 (38.19)
	Task	2,44	27.6	1.15E–6	0.557	aud (40.52) > vis (40.02) > mot (36.40)
	Prescan	1,22	14.1	0.001	0.391	OFF (40.18) > ON (37.78)
	Coil × task	2,44	4.03	0.030	0.155	
	Coil × prescan	1,22	36.1	4.69E–6	0.622	
	Task × prescan	2,44	1.08	0.345	0.047	
	Coil × task × prescan	2,44	1.34	0.270	0.058	
Motor	Coil	1,22	0.02	0.888	0.001	
	Task	2,44	23.2	3.87E–6	0.514	aud (46.38) > vis (45.64) > mot (41.89)
	Prescan	1,22	159	1.47E–11	0.879	OFF (52.12) > ON (37.16)
	Coil × task	2,44	4.13	0.028	0.158	
	Coil × prescan	1,22	29.5	1.84E–5	0.573	
	Task × prescan	2,44	1.31	0.280	0.056	
	Coil × task × prescan	2,44	2.30	0.116	0.095	
Auditory	Coil	1,22	9.81	0.005	0.309	64-channel (39.00) > 20-channel (38.84)
	Task	2,44	25.7	2.94E–6	0.539	aud (39.46) > vis (38.73) > mot (35.58)
	Prescan	1,22	74.4	1.65E–8	0.772	ON (40.44) > OFF (35.41)
	Coil × task	2,44	3.17	0.060	0.126	
	Coil × prescan	1,22	1.47	0.237	0.063	
	Task × prescan	2,44	2.70	0.081	0.109	
	Coil × task × prescan	2,44	1.05	0.356	0.046	
Thalamus	Coil	1,22	9.85	0.005	0.309	20 (34.11) > 64- (32.28)
	Task	2,44	24.9	3.53E–6	0.532	aud (34.41) > vis (34.05) > mot (31.13)
	Prescan	1,22	1323	3.78E–21	0.984	ON (42.09) > OFF (24.30)
	Coil × task	2,44	1.71	0.195	0.072	
	Coil × prescan	1,22	99.7	1.23E–9	0.819	
	Task × prescan	2,44	7.81	0.002	0.262	
	Coil × task × prescan	2,44	0.09	0.896	0.004	

***(A)** Results of the four-factorial ANOVA and **(B)** follow-up ANOVAs separated for each ROI. tSNR, time-course signal-to-noise ratio; ROI, region of interest. vis = visual task/ROI, aud = auditory task/ROI, mot = motor task/RO.*

**FIGURE 6:**
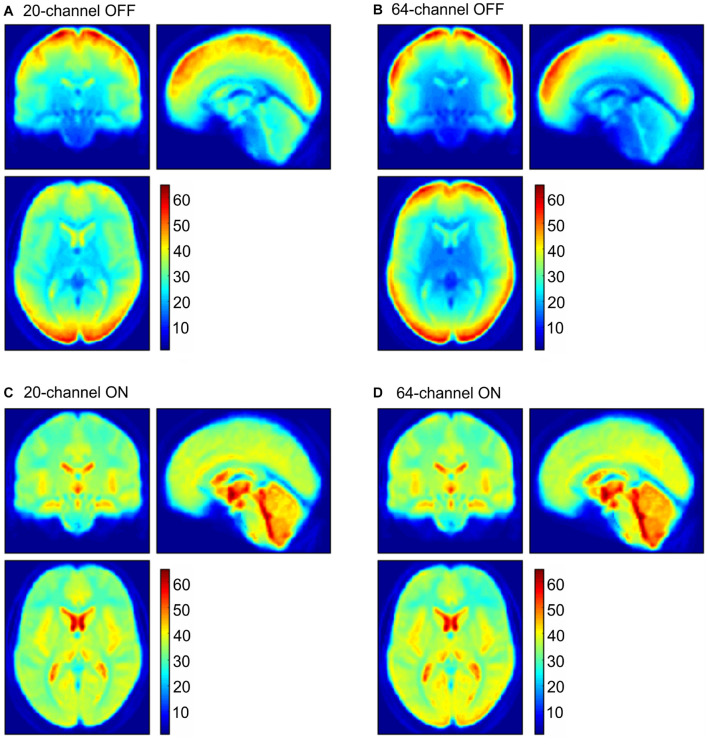
tSNR maps for the 20-channel head coil (**A,C** left column) and the 64-channel head coil (**B,D** right column) with the prescan normalize filter OFF (**A,B** upper row) and ON (**C,D** lower row). tSNR, time-course signal-to-noise ratio.

In addition to ROI analyses, the normalized distribution of the tSNR over the whole brain is shown in Figure 6 for the four possible head coil and prescan normalize conditions.

### Structural Data

#### MRIQC for Structural Data

**FIGURE 7:**
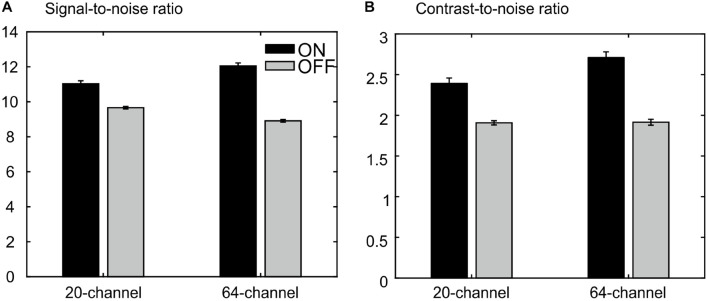
MRIQC results for the structural data: **(A)** results for the analyses of the signal-to-noise ratio, larger values are better. **(B)** Results of analyses of the contrast-to-noise ratio; larger values are better. There is an advantage for the prescan normalize filter turned ON.

The ANOVA for the SNR revealed a main effect of prescan normalize, F(1,25) = 302, *p* = 1.78E–15, η^2^ = 0.923, with larger SNR for prescan normalize ON, as well as an interaction head coil × prescan normalize, F(1,25) = 113, *p* = 8.63E–11, η^2^ = 0.818, with larger SNR differences regarding prescan normalize in the 64-channel head coil, but no main effect of head coil, F(1,25) = 2.21, *p* = 0.148, η^2^ = 0.081 (Figure 7A).

The ANOVA for CNR showed a main effect of head coil, F(1,25) = 17.8, *p* = 2.80E–4, η^2^ = 0.416, with larger values for the 64-channel head coil, a main effect of prescan normalize, F(1,25) = 115, *p* = 7.23E–11, η^2^ = 0.822, with larger values with prescan normalize ON, and an interaction head coil × prescan normalize, F(1,25) = 17.5, *p* = 3.06E–4, η^2^ = 0.412, with larger differences regarding prescan normalize in the 64-channel head coil with larger CNR for prescan normalize ON (Figure 7B).

There is almost no difference between head coils without the prescan normalize filter. However, we observed an improvement for the 64-channel had coil with the prescan normalize filter turned ON. Thus, for structural measurements, the use of the 64-channel head coil with the prescan normalize filter ON is recommended.

## Discussion

The goal of this study was a comparison of signal quality between two commercially available head coils and the consequences of the prescan normalize filter with commonly used fMRI settings. In addition, the signal quality of the functional and structural images was evaluated using the MRIQC tool. Results of the MRIQC tool with respect to the functional data indicate a better performance for the 20-channel head coil with the prescan normalize filter ON. However, with regard to the fMRI analyses in group studies, this recommendation seems to strongly depend on the task and the ROI.

In contrast to the previous studies (Kahn et al., 2007; Kaza et al., 2011) where only one task was used, in the current study, three different tasks and corresponding ROIs were used. In general, the ROI × task interaction indicated that the tasks worked as expected; e.g., there were larger β estimates in the visual cortex during the visual task, and the same holds true for the auditory and motor tasks. Kaza et al. (2011) and Kahn et al. (2007) reported higher activations with the 32-channel head coil compared with the 12-channel head coil, mainly in the cortical regions, but not in subcortical ones. Similarly, here, larger β estimates occurred in the 64-channel compared with the 20-channel head coil, but mainly in the visual cortex. Thus, the choice of the head coil itself will not necessarily result in a significant difference in the results. However, there was an effect of the prescan normalize filter. In the auditory cortex and the thalamus, larger β estimates were found for the prescan normalize filter ON, whereas in the motor and visual cortices, β estimates were larger for prescan normalize OFF, independent of the choice of the head coil. In contrast to Kaza et al. (2011), who found that filtering did not affect the β estimates in the 12-channel but in the 32-channel head coil mainly in subcortical and cerebellar areas, in the current study, the effect of enhanced activation in the subcortical areas occurred in both head coils with the prescan normalize filter ON. However, enhanced activation was found in the visual and motor cortices with the prescan normalize filter OFF. Thus, summarizing the results of the β estimates, the choice of the head coil may not lead to significant differences within the data, with the exception of the visual cortex. However, the choice of turning ON or OFF the prescan normalize filter might indeed affect the data. In case researchers are interested in the more central parts or the auditory cortex, it is recommended to use the filter, whereas it is not necessary for studies interested in motor and visual cortices. In summary, we were able to expand the findings by Kaza et al. (2011) in two additional head coils and additional auditory and visual tasks targeting brain areas with wider range of distances between brain and coils.

In addition to the β estimates, tSNR differences between head coils and prescan normalize filter also depend on the task and ROI. In line with the previous results, the tSNR in the auditory cortex and thalamus was higher with prescan normalize ON, whereas tSNR was higher in the areas closer to the coil elements, such as visual and motor cortices, with prescan normalize OFF. Regarding the differences in the head coils, the tSNR in the visual cortex was higher in the 64-channel head coil. The same pattern occurred for the auditory cortex, whereas the thalamus showed higher tSNR with the 20-channel head coil. The overall pattern of the tSNR underlined the findings regarding the prescan normalize filter, where it was clearly visualized, that in the central parts, the tSNR was higher with the prescan normalize ON, whereas in the cortex, the prescan normalize OFF resulted in higher tSNR. Previous studies found an increase in (t)SNR with head coils with more channels, especially in multislice accelerated measurements (Seidel et al., 2020), which was not done in the current study, where only in-plane acceleration (GRAPPA = 2) was used, or in the subcortical areas (Kaza et al., 2011). In our study, however, except for the visual cortex, which is closest to the coil elements, there was no clear improvement in tSNR with respect to the 64-channel head coil. However, there was a clear difference when using the prescan normalize filter, which was not investigated by the previous studies.

As the prescan normalize filter is not only a scalar multiplication of signal intensities, but a complex-values multiplication of smoothed images, it is able to change (t)SNR as well as β estimates. One explanation might be because the prescan normalize filter only compensates for the RF field inhomogeneities of the receiving coils (here, the surface coils), as described above, by assuming a homogeneous sensitivity of the body coil. However, some inhomogeneity in the body coil might still exists, especially present as signal intensity loss toward the edges. Furthermore, Lutti et al. (2010) showed that local flip angle variations caused by RF transmit inhomogeneities especially in 3T are stronger in the cortical than subcortical regions. The prescan normalize filter does not account for such irregularities, which might affect the differences in the activation strength, i.e., the β estimates, especially in the subcortical regions as shown in the current study and described by Kaza et al. (2011).

Besides the analyses of the functional data, we also analyzed the structural data measured with the two different head coils and with the prescan normalize filter. The results of the MRIQC tool indicated the best results in almost all parameters for the 64-channel head coil with the prescan normalize filter turned ON.

Results of the MRIQC tool, which did not account for any activation patterns but rely on the raw data, indicated a better performance with the 20-channel head coil for functional measurements and the 64-channel head coil for anatomical measurements, both with the use of the prescan normalize filter. Nevertheless, these results might not be generalizable for all MRI experiments. It may depend on the sequences and their parameters; e.g., when using a multislice protocol, with different acceleration methods or different voxel sizes, the performance of the head coils might differ. Seidel et al. (2020) compared both the 20-channel and 64-channel head coils with various acceleration methods and found that tSNR losses occurred more in the 20-channel head coil than in the 64-channel head coil while enhancing the acceleration factor. Summarizing the results of the current study, we could show that in general there was no large difference between the 20-channel and 64-channel head coils when performing fMRI paradigms. However, depending on the ROI, especially, in the visual cortex, the 64-channel head coil might have some advantages. The most important result is the influence of the prescan normalize filter. For studies interested in the subcortical brain areas and the auditory cortex, the usage of the prescan normalize filter is recommended, whereas it seems not necessary in the visual and motor cortices.

## Data Availability Statement

The datasets generated for this study are available on request to the corresponding author.

## Ethics Statement

The studies involving human participants were reviewed and approved by Kommission für Forschungsfolgenabschätzung und Ethik, Carl-von-Ossietzky Universität Oldenburg. The participants provided their written informed consent to participate in this study.

## Author Contributions

TS and JR contributed to conception and design of the study and the interpretation of the results. TS was responsible for the data collection and analyses and wrote the first draft of the manuscript. Both authors contributed to manuscript revision and read and approved the submitted version.

## Conflict of Interest

The authors declare that the research was conducted in the absence of any commercial or financial relationships that could be construed as a potential conflict of interest.

## Publisher’s Note

All claims expressed in this article are solely those of the authors and do not necessarily represent those of their affiliated organizations, or those of the publisher, the editors and the reviewers. Any product that may be evaluated in this article, or claim that may be made by its manufacturer, is not guaranteed or endorsed by the publisher.

## Footnotes

1
https://doi.org/10.1038/s41597-019-0035-4

2
https://github.com/CPernet/spmup/blob/master/QA/spmup_temporalSNR.m
