# Supplementary material for: Recommendations of Choice of Head Coil and Prescan Normalize Filter Depend on Region of Interest and Task
Source: Front Neurosci. 2021 Oct 29;15:735290. doi: 10.3389/fnins.2021.735290 (PMC8585748; doi:10.3389/fnins.2021.735290)
Supplement: Supplementary file 1 [file Data_Sheet_1.docx]

Supplementary Material

# MRIQC BOLD

The image quality metrics for the functional images were separated into measures for the spatial information, measures for the temporal information and measures for artifacts and other. Mean values can be found in Supplementary Table 1.

## Measures for the spatial information

Measures for the spatial information are the entropy focus criterion (EFC), the foreground-to-background energy ratio (FBER), the full-width-half-maximum (FWHM), the signal-to-noise ratio (SNR) as well as the summary statistics. The EFC describes the entropy of voxel intensities as an indication of ghosting and blurring induced by head motion (1), i.e. lower values are better. The ANOVA revealed a main effect of head coil, F(1,25) = 122.24, p < .001, η² = .830, with lower values for the 20-channel head coil, a main effect of prescan normalize, F(1,25) = 108.30, p < .001, η² = .812, with lower values for prescan normalize ON and an interaction between head coil and prescan normalize, F(1,25) = 54.08, p < .001, η² = .684. In addition, there was a main effect of task, F(2,50) = 6.30, p = .003, η² = .202, with slightly lower values for the auditory task as well as an interaction between head coil and task, F(2,50) = 10.90, p < .001, η² = .303.

The FBER is defined as the mean energy of image values within the head relative to the outside. Higher values are better. The ANOVA revealed a main effect of head coil, F(1,25) = 168.68, p < .001, η² = .821, with higher values for the 20-channel head coil, a main effect of prescan normalize, F(1,25) = 2026.65, p < .001, η² = .987, with higher values for prescan normalize ON and an interaction between head coil and prescan normalize, F(1,25) = 5.31, p = .03, η² = .175. In addition, there was an interaction between head coil and task, F(2,50) = 8.31, p < .001, η² = .242, with overall higher values for the 20-channel head coil decreasing from motor to auditory and visual task, whereas in the 64-channel head coil, the visual and auditory task had higher values than the motor task.

The ANOVA for the SNR, showed a main effect of head coil, F(1,25) = 164.18, p < .001, η² = .868, with larger values for the 20-channel head coil and a main effect of prescan normalize, F(1,25) = 2616.41, p < .001, η² = . 990, with better SNR for prescan normalize ON. There was a task x prescan normalize interaction, F(2,50) = 3.74, p = .03, η² = .130, with no difference in prescan normalize OFF, but an increase in SNR from motor to auditory and visual task.

For the FWHM smoothness, which is the spatial distribution of the image intensity values in units of voxels, lower values are better. The ANOVA revealed a main effect of head coil, F(1,25) = 9.19, p = .005, η² = .269, with lower values in the 20-channel head coil, a main effect of prescan normalize, F(1,25) = 67.00, p < .001, η² = .729, with lower values for prescan normalize ON and a main effect of task, F(2,50) = 4.27, p = .019, η² = .145, with lowest values in the visual task.

The summary statistics calculate values for the background and foreground which show contrasting results. The results for the background statistics showed a main effect of head coil, F(1,25) = 133.79, p < .001, η² = .842, with lower values for the 20-channel head coil, a main effect of prescan normalize, F(1,25) = 903.26, p < .001, η² = .973, with lower values for prescan normalize ON and an interaction between head coil and prescan normalize, F(1,25) = 101.71, p < .001, η² = .803, with larger differences between prescan normalize ON and OFF in the 20-channel, compared to the 64-channel head coil. In addition, there was a main effect of task, F(2,50) = 4.38, p = .017, η² = .149, with lowest values in the motor followed by the auditory and visual task. For the foreground, the ANOVA revealed a main effect of head coil, F(1,25) = 24.74, p < .001, η² = .497, with lower values in the 64-channel head coil, a main effect of prescan normalize, F(1,25) = 29.92, p < .001, η² = .544, with lower values for prescan normalize ON and an head coil x prescan normalize interaction, F(1,25) = 276.37, p < .001, η² = .917, with an increase from prescan normalize ON to OFF in the 20-channel head coil, whereas there was a decrease in the 64-channel head coil. There was also a main effect of task, F(2,50) = 3.75, p = .03, η² = .130, with an increase from visual to auditory and motor task as well as an task x prescan normalize, F(2,50) = 4.94, p = .02, η² = .143, and task x prescan normalize x head coil interaction, F(2,50) = 4.94, p = .01, η² = .165.

Summarizing the measures of spatial information recommend the usage of the 20-channel head coil with prescan normalize ON.

## Measures of temporal information

The temporal derivative (DVARS) of time courses with the root-mean-square variance over voxels indexes the rate of change of BOLD signal across the entire brain at each frame of data and is calculated after motion correction. Intensities are scaled to 1000 leading to the units being expressed in x10 %ΔBOLD change. The DVARS metric is normalized with the standard deviation of the temporal difference time series. The ANOVA only showed significant main effects of head coil, F(1,25) = 4.75, p = .03, η² = .160, with lower values for the 20-channel head coil, a main effect of prescan normalize, F(1,25) = 165.15, p < .001, η² = .868, with lower values for prescan normalize OFF and a main effect of task, F(2,50) = 6.31, p = .003, η² = .201, with lower values for the motor task.

The global correlation (GCOR) calculates an optimized summary of time-series correlation as in (2) and illustrates the differences between data due to motion / physiological noise / imaging artifacts. Values closer to zero are better. The ANOVA revealed a main effect of head coil, F(1,25) = 6.51, p = .017, η² = .206, lower values for the 20-channel head coil, a main effect of prescan normalize, F(1,25) = 26.17, p < .001, η² = .511, with lower values for prescan normalize ON and a main effect of task, F(2,50) = 5.98, p = .004, η² = .193, with lower values in the auditory task.

The temporal SNR is a simplified interpretation of the tSNR definition by (3). They report the median value of the tSNR map calculated as the quotient from the average BOLD signal across time and the corresponding temporal standard-deviation map. The ANOVA only showed a main effect of task, F(2,50) = 13.59, p = .001, η² = .352, with increasing tSNR from motor to visual and auditory task.

Similar to the measures of spatial information, also the measures of temporal information recommend the usage of the 20-channel head coil together with the prescan normalize filter ON.

## Measures of artifacts

As measures of artifacts MRIQC provides the framewise displacement, the ghost-to-signal ratio, the AFNI’s outlier ratio as well as the AFNI’s quality index. The framewise displacement expresses instantaneous head-motion, whereby rotational displacements are calculated as the displacement on the surface of a sphere of radius 50 mm according to (4). There was a main effect of head coil, F(1,25) = 5.74, p = .02, η² = .186, with lower values in the 64-channel head coil and an interaction of head coil x prescan normalize, F(1,25) = 7.3, p = .011, η² = .227, with lower values in the 64-channel head coil and a larger difference between prescan normalize ON and OFF in the 20-channel head coil.

The ghost-to-signal ratio (GSR) is calculated along the two possible phase-encoding axis x and y. The ANOVA for the GSR in the x direction, results in a main effect of head coil, F(1,25) = 12.32, p = .001, η² = .330, with lower in the 20-channel head coil, a main effect of prescan normalize, F(1,25) = 7.75, p = .01, η² = .236, with lower values with prescan normalize ON, as well as the interaction between head coil and prescan normalize, F(1,25) = 43.89, p < .001, η² = .637, larger differences in prescan normalize OFF. In addition there was a task x prescan normalize interaction, F(1,25) = 3.8, p = .02, η² = .132, with lowest values in the visual task. In the y-direction, there was also the main effect of head coil, F(1,25) = 27.95, p < .001, η² = .527, with lower values in the 20-channel head coil and a main effect of prescan normalize, F(1,25) = 87.26, p < .001, η² = .777, with lower values in prescan normalize ON as well as an interaction between head coil and prescan normalize, F(1,25) = 8.8, p = .006, η² = .261.

Finally, there are two measures calculated with AFNI, the outlier ratio (AOR) and the quality index (AQI). The AOR is the mean fraction of outliers per fMRI volume as given by AFNI’s 3dToutcount. The ANOVA did not show any significant effects. The AQI represents the mean quality index as computed by AFNI’s 3dTqual. The ANOVA only showed a main effect of head coil, F(1,25) = 20.79, p < .001, η² = .454, with larger values for the 20-channel head coil, a main effect of prescan normalize, F(1,25) = 323.64, p < .001, η² = .928, with larger values for prescan normalize ON and an interaction between head coil x prescan normalize, F(1,25) = 10.47, p = .003, η² = 295. There was also a main effect of task, F(2,50) = 4.17, p = .02, η² = .143.

All three reported types of measures lead to the conclusion that the 20-channel head coil is better suited for fMRI experiments than the 64-channel head coil, together with the prescan normalize filter turned on.

|  | 20-channel | | | | | | | 64-channel | | | | | | | BEST | |
| --- | --- | --- | --- | --- | --- | --- | --- | --- | --- | --- | --- | --- | --- | --- | --- | --- |
|  | Motor | | Auditory | | Visual | | Motor | | | Auditory | | Visual | |  | |  |
|  | ON | OFF | ON | OFF | ON | OFF | ON | | OFF | ON | OFF | ON | OFF |  | |  |
| Measures of spatial information | | | | | | | | | | | | | | | | |
| EFC | 0.558 | 0.567 | 0.559 | 0.568 | 0.559 | 0.569 | 0.565 | | 0.588 | 0.565 | 0.587 | 0.559 | 0.588 | 20 ON | |  |
| FBER | 1819 | 675 | 1804 | 672 | 1785 | 670 | 1449 | | 268 | 1499 | 281 | 1509 | 280 | 20 ON | |  |
| SNR | 4.93 | 2.92 | 4.96 | 2.92 | 4.97 | 2.93 | 4.48 | | 2.38 | 4.48 | 2.37 | 4.51 | 2.36 | 20 ON | |  |
| FHWM | 2.379 | 2.486 | 2.368 | 2.487 | 2.367 | 2.482 | 2.419 | | 2.506 | 2.413 | 2.399 | 2.401 | 2.502 | 20 ON | |  |
| BG | 34.3 | 54.2 | 34.6 | 54.7 | 34.8 | 55.1 | 39.9 | | 64.8 | 39.7 | 64.8 | 39.9 | 65.1 | 20 ON | |  |
| FG | 760 | 863 | 758 | 862 | 758 | 862 | 793 | | 736 | 790 | 739 | 788 | 737 | 64 ON | |  |
| Measures of temporal information | | | | | | | | | | | | | | | | |
| DVARS | 1.207 | 1.107 | 1.223 | 1.108 | 1.220 | 1.135 | 1.221 | | 1.100 | 1.249 | 1.137 | 1.255 | 1.136 | 20 OFF | |  |
| GCOR | 0.017 | 0.022 | 0.013 | 0.018 | 0.015 | 0.021 | 0.023 | | 0.023 | 0.014 | 0.023 | 0.019 | 0.024 | 20 ON | |  |
| tSNR | 51.4 | 53.0 | 55.8 | 54.8 | 54.8 | 54.3 | 51.9 | | 51.6 | 57.2 | 58.6 | 57.8 | 56.2 | - | |  |
| Measures of artifacts | | | | | | | | | | | | | | | | |
| FD | 0.13 | 0.13 | 0.13 | 0.16 | 0.16 | 0.17 | 0.14 | | 0.13 | 0.13 | 0.12 | 0.13 | 0.13 | 64 | |  |
| GSRx | 0.0019 | 0.0013 | 0.0018 | 0.0007 | 0.0017 | 0.0010 | 0.0012 | | 0.0093 | 0.0012 | 0.0088 | 0.0008 | 0.0087 | 20 ON | |  |
| GSRy | 0.0209 | 0.0306 | 0.0213 | 0.0312 | 0.0214 | 0.0312 | 0.0248 | | 0.0382 | 0.0245 | 0.0382 | 0.0246 | 0.0390 | 20 ON | |  |
| AOR | 0.002 | 0.002 | 0.002 | 0.002 | 0.003 | 0.004 | 0.003 | | 0.002 | 0.002 | 0.001 | 0.002 | 0.002 | - | |  |
| AQI | 0.010 | 0.003 | 0.009 | 0.003 | 0.009 | 0.003 | 0.008 | | 0.003 | 0.008 | 0.002 | 0.006 | 0.003 | 20 ON | |  |

Supplementary Table 1: Mean values of the different measures separated for each type of measure (spatial information, temporal information and artifacts) and each condition, i.e. head coil (20-channel vs. 64-channel), task (motor, auditory or visual) and prescan normalize (ON vs. OFF). The last column indicated which combination of head coil and prescan normalize filter is recommended the best according to the results of the ANOVA.

# MRIQC T1

The image quality metrics for the structural images were separated into measures of noise measurements, measures based on information theory, specific artifacts and other. Mean values for the different measurements separated for conditions can be found in Supplementary Table 2.

## Measures of noise measurements

The MRIQC tool provides four different noise measurements. The signal-to-noise ratio (SNR) is calculated according to (1), using the air background as noise reference. Additionally, for images that have undergone some noise reduction processing, or the more complex noise realization of current parallel acquisitions, a simplified calculation using the within tissue variance is also provided. MRIQC separates the results into grey matter, white matter and cerebrospinal fluid and the overall SNR. Since the results are comparable and point to the same pattern, only the results of the ANOVA for the overall SNR are reported. There was a main effect of head coil, F(1,25) = 71.88, p < .001, η² = .742, with higher SNR for the 64-channel head coil, a main effect of prescan normalize, F(1,25) = 288.93, p <.001, η² = .920, with higher SNR for prescan normalize ON, as well as an interaction between head coil and prescan normalize, F(1,25) = 47.34, p <.001, η² = .654, with higher SNR differences between the prescan normalize ON and OFF in the 64-channel head coil. The same pattern is seen for the different tissues separately.

The contrast-to-noise ratio (CNR) (2) is an extension of the SNR calculation to evaluate how separated the tissue distributions of grey and white matter are. Therefore, higher values indicate better signal quality. The ANOVA showed a main effect of head coil, F(1,25) = 17.81, p < .001, η² = .416, with higher values for the 64-channel head coil, a main effect of prescan normalize, F(1,25) = 115.67, p < .001, η² = .822, with higher values for prescan normalize ON, and an interaction between head coil and prescan normalize, F(1,25) = 17.53, p < .001, η² = .412, with higher differences between prescan normalize ON and OFF in the 64-channel head coil.

In the coefficient of joint variation for the of grey and white matter (3) higher values are related to the presence of heavy head motion and large intensity non-uniformity artifacts. Since there is no obvious head motion artefact in the visual inspection of the data, the results seem to relay on the intensity non-uniformity artifacts. The ANOVA showed in a main effect of head coil, F(1,25) = 27.94, p < .001, η² = .527, with lower values for the 64-channel head coil, a main effect of prescan normalize, F(1,25) = 174.80, p <.001, η² = .875, with lower values for prescan normalize ON as well as a significant interaction between head coil and prescan normalize, F(1,25) = 11.21, p = .002, η² = .309, with larger difference between the prescan normalize ON and OFF in the 64-channel head coil.

The Mortamet’s quality index 2 (QI2) (4) is a calculation of the goodness-of-fit of the Χ² distribution on the air mask once the artifactual intensities detected for computing the QI1 (see measures of specific artifacts) index have been removed, in other words, a noise distribution analysis. Results of the ANOVA showed a main effect of head coil, F(1,25) = 19.59, p < .001, η² = .439, with higher values for the 64-channel head coil, a main effect of prescan normalize, F(1,25) = 147.26, p < .001, η² = .854, with higher values for prescan normalize ON and an interaction between head coil and prescan normalize, F(1,25) = 10.22, p = .003, η² = .290, with differences between prescan normalize ON and OFF only in the 64-channel head coil.

Summarizing, all the measures of noise measurement recommend the usage of the 64-channel head coil with the prescan normalize filter ON.

## Measures based on information theory

There are two measures based on information theory: the entropy focus criterion (EFC) and the foreground-to-background energy ratio (FBER). The EFC described by (5) which uses the Shannon entropy of voxel intensities as an indicator of ghosting and blurring induced by head movement, this means that lower values are better. The results of the ANOVA showed a main effect of head coil, F(1,25) = 78.47, p < .001, η² = .758, with lower values for the 64-channel head coil, a main effect of prescan normalize, F(1,25) = 16.87, p < .001, η² = .403, with lower values for the prescan normalize ON and an interaction between head coil and prescan normalize, F(1,25) = 230.86, p < .001, η² = .902, with an increase from prescan normalize ON compared to OFF in the 20-channel head coil and a decrease in the 64-channel head coil.

The FBER (6) is defined as the mean energy value of image values within the head relative to outside of the head, i.e. higher values are better. The ANOVA showed a main effect of head coil, F(1,25) = 205.19, p < .001, η² = .891, with higher values for the 64-channel head coil and a main effect of prescan, F(1,25) = 931.15, p < .001, η² = .974, with higher values for prescan normalize ON and an interaction between head coil and prescan normalize, F(1,25) = 285.31, p < .001, η² = .919, indicating a larger difference between prescan normalize ON compared to OFF and overall higher values in the 64-channel head coil compared to the 20-channel.

Both measures based on information theory lead to the recommendation to use the 64-channel head coil with the prescan normalize filter ON.

## Specific artifacts

There are several parameters targeting specific artifacts in the MRIQC tool. The first one is the intensity non-uniformity (7) which characterizes a summary statistic of the intensity non-uniformity field as extracted by the N4ITK algorithm proposed by (7). This measures the location and spread of the bias field extracted estimated by the intensity non-uniformity correction. The smaller spreads located around 1.0 are better. The ANOVA showed a main effect of head coil, F(1,25) = 33.44, p < .001, η² = .572, with values closer to one for the 64-channel head coil and a main effect of prescan, F(1,25) = 730.31, p < .001, η² = .967, with values closer to one for prescan normalize OFF.

The second one detects artifacts in the image using the method described in (4). The quality index QI1 is the proportion of voxels with intensity corrupted by artifacts normalized by the number of voxels in the background. It measures the amount of artifactual intensities in the air surrounding the head above the nasio-cerebellar axis, thus, the smaller QI1 the better. However, the ANOVA with this parameter did not show any significant main effect or interaction. This means on the other hand, that there are no severe or systematic artifacts within the data.

Another measure is the white matter to maximum intensity ratio which is defined as the median intensity within the white matter mask over the 95% percentile of the full intensity distribution, that captures the existence of long tails due to hyper-intensity of the carotid vessels and fat. Values should be around the interval 0.6 to 0.8. However, our values are not in the specified range, in fact between 0.3 and 0.4 which might lead to the conclusion that we have less contrast than expected. The ANOVA revealed a main effect of head coil, F(1,25) = 175.50, p < .001, η² = .875, with higher values for the 20-channel coil, a main effect of prescan normalize, F(1,25) = 301.37, p < .001, η² = .923, with higher values for prescan normalize ON as well as an interaction between head coil and prescan normalize, F(1,25) = 121.65, p < .001, η² = .829, with larger difference between prescan normalize ON and OFF in the 64-channel head coil.

## Other measures

Furthermore, there are some more not further specified measures, as e.g. the intra-cellular volume fractions (ICVS) of each tissue calculated on the FSL FASTs segmentation. Normative values fall around 20%, 45% and 35% for cerebrospinal fluid, white and grey matter, respectively. Within our dataset, the mean values are 16%, 40% and 44% respectively. The results of the different ANOVAs for each tissue did not show the same pattern. Whereas for cerebrospinal fluid, there was a weak main effect of head coil, F(1,25) = 7.63, p = .01, η² = .233, with slightly higher values in the 64-channel, a main effect of prescan normalize, F(1,25) = 174.20, p < .001, η² = .874, with larger values for prescan normalize ON as well as an interaction head coil x prescan normalize, F(1,25) = 53.11, p < .001, η² = .680, with larger differences in the 64-channel head coil. The ICVS for white matter showed a contrary pattern with a main effect of head coil, F(1,25) = 16.44, p < .001, η² = .397, with slightly higher values for the 20-channel and a main effect of prescan normalize, F(1,25) = 20.68, p < .001, η² = .453, with slightly higher values for the prescan normalize OFF but no interaction. Further, for grey matter, there was no main effect of head coil, but a main effect of prescan normalize, F(1,25) = 62.80, p < .001, η² = .715, with higher values for prescan normalize ON and a head coil x prescan normalize interaction, F(1,25) = 38.97, p < .001, η² = .609, with a larger difference between prescan normalize ON and OFF in the 64-channel head coil.

The residual partial volume effect (rPVE) feature is a tissue-wise sum of partial volumes that fall in the range [5%-95%] of the total volume of a pixel, computed on the partial volume maps generated by FSL FAST. Smaller rPVEs are better. The ANOVA for grey matter showed a main effect of head coil, F(1,25) = 21.67, p < .001, η² = .464, with smaller values for the 64-channel head coil, a main effect of prescan normalize, F(1,25) = 501.77, p < .001, η² = .953, with smaller values for the prescan normalize OFF as well as an interaction head coil x prescan normalize, F(1,25) = 119.40, p < .001, η² = .827, with larger differences between prescan normalize ON and OFF in the 64-channel head coil. The ANOVA for white matter did not show a main effect of head coil, but a main effect of prescan normalize, F(1,25) = 800.64, p < .001, η² = .970, with smaller values in prescan normalize OFF and an interaction head coil x prescan normalize, F(1,25) = 25.33, p < .001, η² = .503, with larger differences between prescan normalize ON and OFF in the 64-channel head coil. For cerebrospinal fluid, there was a main effect of head coil, F(1,25) = 6.70, p = .015, η² = .211, with smaller values for the 64-channel head coil, a main effect of prescan normalize, F(1,25) = 122.23, p < .001, η² = .830, with smaller values for the prescan normalize ON, as well as an interaction head coil x prescan normalize, F(1,25) = 37.52, p < .001, η² = .600, with larger differences between prescan normalize ON and OFF in the 64-channel head coil.

The full-width-half-maximum (FWHM) measure describes the spatial distribution of the image intensity values in units of voxels (8), thus the blurriness of the image where lower values are better. This measure is calculated for x, y, and z direction as well as an average over all directions. Because the patterns looked similar only the averaged measure was reported. The ANOVA results in a main effect of head coil, F(1,25) = 293.54, p < .001, η² = 9.21, with lower values for the 20-channel head coil, a main effect of prescan normalize, F(1,25) = 1159.3, p <.001, η² = .979, with lower values for the prescan normalize ON as well as an interaction head coil x prescan normalize, F(1,25) = 181.35, p < .001, η² = .879, where the difference between prescan normalize ON and OFF is smaller in the 20-channel head coil. Whereas the previous ones did not show a clear recommendation, because the pattern look different for the different tissues, FWHM leads to the usage of the 20-channel head coil with the prescan normalize filter ON.

Finally, several summary statistics are computed for background, grey matter, white matter and cerebrospinal fluid, estimating the mean, standard deviation as well as the 95% and 5% percentiles of each tissue distribution. The ANOVA for the mean in the background revealed a main effect of head coil, F(1,25) = 36.12, p < .001, η² = .591, with smaller values for the 64-channel head coil and a main effect of prescan normalize, F(1,25) = 883.48, p < .001, η² = .972, with smaller values for prescan normalize ON. For grey matter, there was a main effect of head coil, F(1,25) = 26.73, p < .001, η² = .517, with smaller values for the 64-channel head coil and a head coil x prescan normalize interaction, F(1,25) = 5.63, p = .026, η² = .183, whereby the was an increase from prescan normalize ON to OFF in the 20-channel head coil, whereas there was a decrease in the 64-channel head coil. For white matter, there was a main effect of head coil, F(1,25) = 13.65, p = .001, η² = .353, with smaller values for the 20-channel head coil, a main effect of prescan normalize, F(1,25) = 121.41, p < .001, η² = .829, with smaller values for the prescan ON and an interaction head coil x prescan normalize, F(1,25) = 46.29, p < .001, η² = .649, with larger differences between prescan normalize ON compared to OFF in the 64-channel head coil. For cerebrospinal fluid none of the main effects or interactions was significant.

Overall, most IQMs in the T1 evaluation using the MRIQC tool indicate better results for the 64-channel head coil with the prescan normalize filter ON.

|  | 20-channel | | 64-channel | | Best |
| --- | --- | --- | --- | --- | --- |
|  | ON | OFF | ON | OFF |  |
| Measures of noise measurements | | | | | |
| SNRd | 58.9 | 19.6 | 104.4 | 25.3 | 64 ON |
| CNR | 2.391 | 1.908 | 2.709 | 1.915 | 64 ON |
| CJV | 0.571 | 0.716 | 0.481 | 0.707 | 64 ON |
| QI2 | 0.005 | 0.002 | 0.007 | 0.002 | 64 ON |
| Measures based on information theory | | | | | |
| EFC | 0.600 | 0.615 | 0.584 | 0.580 | 64 ON |
| FBER | 1229 | 375 | 3449 | 550 | 64 ON |
| Specific artifacts | | | | | |
| INU_range | 0.616 | 0.867 | 0.687 | 0.922 | 64 OFF |
| QI1 | 3.0e-5 | 1.8e-5 | 2.4e-4 | 1.9e-4 | - |
| wm2max | 0.427 | 0.354 | 0.409 | 0.270 | 20 ON |
| Other measures | | | | | |
| ICVS - GM | 0.435 | 0.440 | 0.427 | 0.450 | ON |
| ICVS - WM | 0.404 | 0.441 | 0.397 | 0.404 | 20 OFF |
| ICVS - CSF | 0.161 | 0.149 | 0.175 | 0.146 | 64 ON |
| rPVE - GM | 11.26 | 10.72 | 11.38 | 10.37 | 64 OFF |
| rPVE - WM | 13.74 | 12.59 | 13.97 | 12.31 | OFF |
| rPVE - CSF | 27.24 | 29.64 | 25.48 | 29.90 | 64 ON |
| FHWM | 3.93 | 4.71 | 4.32 | 5.59 | 20 ON |
| BG | 20.7 | 46.4 | 11.8 | 35.1 |  |
| GM | 794 | 803 | 791 | 788 |  |
| WM | 1001 | 1004 | 1000 | 1009 |  |
| CSF | 408 | 417 | 432 | 427 |  |

Supplementary Table 2: Mean values of the different measures separated for each type of measure and each condition, i.e. head coil (20-channel vs. 64-channel) and prescan normalize (ON vs. OFF). The last column indicated which combination of head coil and prescan normalize filter is recommended the best according to the results of the ANOVA
